# Polyketides with Immunosuppressive Activities from Mangrove Endophytic Fungus *Penicillium* sp. ZJ-SY_2_

**DOI:** 10.3390/md14120217

**Published:** 2016-11-25

**Authors:** Hongju Liu, Senhua Chen, Weiyang Liu, Yayue Liu, Xishan Huang, Zhigang She

**Affiliations:** 1 School of Chemistry, Sun Yat-Sen University, Guangzhou 510275, China; liuhj8@mail2.sysu.edu.cn (H.L.); chensh65@mail2.sysu.edu.cn (S.C.); liuyayue@mail2.sysu.edu.cn (Y.L.); 2 School of Pharmacy, Guangdong Medical University, Dongguan 523808, China; liuwy79@yahoo.com; 3 South China Sea Bio-Resource Exploitation and Utilization Collaborative Innovation Center, Guangzhou 510006, China

**Keywords:** polyketides, *Penicillium* sp., marine fungus, immunosuppressive activity

## Abstract

Nine polyketides, including two new benzophenone derivatives, peniphenone (**1**) and methyl peniphenone (**2**), along with seven known xanthones (**3**–**9**) were obtained from mangrove endophytic fungus *Penicillium* sp. ZJ-SY_2_ isolated from the leaves of *Sonneratia apetala*. Their structures were elucidated on the basis of MS, 1D, and 2D NMR data. Compounds **1**, **3**, **5**, and **7** showed potent immunosuppressive activity with IC_50_ values ranging from 5.9 to 9.3 μg/mL.

## 1. Introduction

The inhibition of the acute rejection which is the main reason for the loss of a graft’s function is the key to the success of organ transplantation. In clinical therapy, the life-long administration of immunosuppressive drugs has become the routine therapeutic regimen to secure allo-transplantation. Although the currently used immunosuppressive drugs such as cyclosporin A (CSA), tacrolimus, mycophenolate mofetil and sirolimus are very effective, they possess serious side effects including nephrotoxicity and neurotoxicity, and the risks of infection, cancer, new onset post-transplant diabetes mellitus, hyperlipidemia and hypertension [1,2,3,4]. So it is urgent to find effective and safe immunosuppressants.

Mangrove endophytic fungi have been demonstrated to be a rich and reliable source of biologically active and chemically novel compounds [5]. In the past decades, our research group had been focusing on the exploration of new bioactive metabolites from mangrove endophytic fungi collected from the South China Sea [6,7,8,9,10,11,12,13]. The EtOAc extract of the fermentation of a mangrove endophytic fungus, *Penicillium* sp. ZJ-SY_2_, which was isolated from the leaves of *Sonneratia apetala*, has been screened to exhibit potent immunosuppressive activity and anti-inflammatory activity. Bioassay-guided fractionation of the EtOAc extract led to the isolation of two new benzophenones derivatives and seven previously reported xanthones. All of the isolated compounds (**1**–**9**) were evaluated for their immunosuppressive activity against concanavalin A (Con A)-induced (T cell) and lipopolysaccharide (LPS)-induced (B cell) proliferations of mouse splenic lymphocytes by the MTT method. Among them, compounds **1**, **3**, **5**, and **7** showed potent immunosuppressive activity. Herein, details of the isolation, structural elucidation, as well as biological activity of these compounds are described.

## 2. Results and Discussion

**Figure 1:**
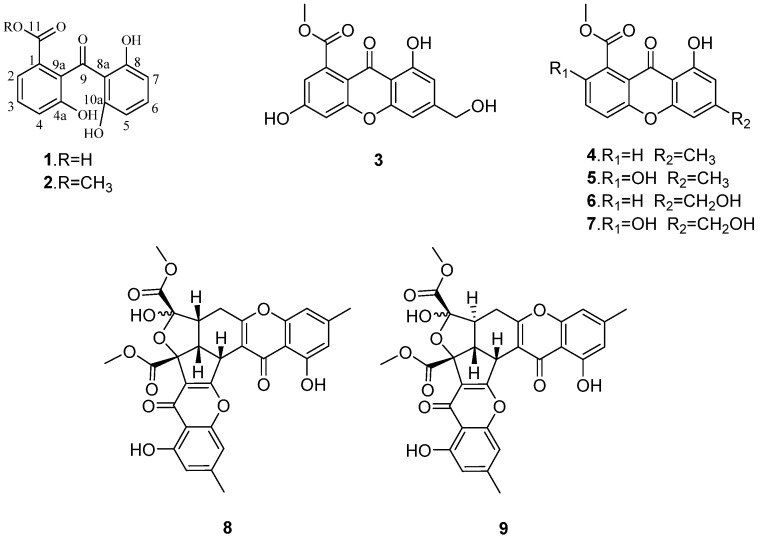
Structures of compounds **1**–**9**.

The mangrove endophytic fungus *Penicillium* sp. ZJ-SY_2_ was cultured on solid rice medium with sea water for four weeks. The EtOAc extract of the fermentation was fractionated by repeated silica gel chromatography and Sephadex LH-20 column chromatography, as well as semi-preparative RP-HPLC, to yield the compounds **1**–**9** (Figure 1). The structure of two new benzophenone derivatives (**1**–**2**) were elucidated on the basis of MS, 1D, and 2D NMR data, and seven known xanthones were identified as conioxanthone A (**3**) [14], methyl 8-hydroxy-6-methyl-9-oxo-9H-xanthene-1-carboxylate (**4**) [15], pinselin (**5**) [16], sydowinin B (**6**) [17], sydowinin A (**7**) [17], remisporine B (**8**) [18], and epiremisporine B (**9**) [18], by comparison of their spectroscopic data with those reported in the literature.

Peniphenone (**1**) was isolated as a yellow solid. The HRESIMS (see Figure S1) displayed a molecular ion peak at *m*/*z* 273.0401 [M − H]^−^ (calcd. for C_14_H_9_O_6_, 273.0405), implying the molecular formula C_14_H_10_O_6_ (10 degrees of unsaturation). Its IR spectrum had absorption bands corresponding to hydroxyl (3354 cm^−1^), carbonyl (1689, 1614 cm^−1^), and aromatic groups (1601, 1500, 1452 cm^−1^). The ^1^H NMR (see Figure S2) data suggesting the presence of two AMX spin systems [δ_H_ 7.25 (t, *J* = 7.9 Hz), 7.49 (d, *J* = 7.7 Hz), and 7.01(d, *J* = 8.1 Hz)] and [δ_H_ 7.19 (t, *J* = 8.2 Hz), 6.28 (d, *J* = 8.2 Hz), and 6.28 (d, *J* = 8.2 Hz)], indicated that **1** possessed two 1,2,3-trisubstituted benzene rings (Table 1). The ^13^C (see Figure S3) and DEPT NMR spectrum resolved the 14 sp^2^-hybridized carbon resonances attributed to one carbonyl function (δ_C_ 203.6, C-9), one carboxyl function (δ_C_ 169.7, C-11), and two aromatic rings (Table 1). The downfield-shift of carbonyl (δ_C_ 203.6, C-9) indicated that **1** possessed a benzophenone framework.

**Figure 2:**
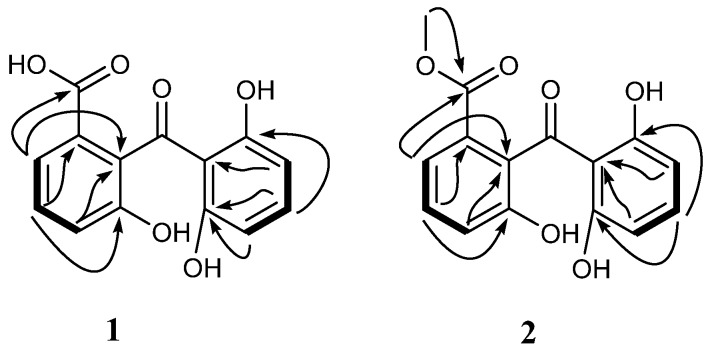
Key HMBC (black arrows) and COSY (black bold lines) correlations of compounds **1** and **2**.

Extensive analysis of 2D NMR (see Figures S4–S6) revealed the structure of compound **1** as described below (Figure 2). The HMBC correlations from the H-2 (δ_H_ 7.49) to the carbonyl carbon (δ_C_ 169.7) and C-9a (δ_C_ 130.4) and the H-3 (δ_H_ 7.25) to C-1(δ_C_ 134.9) indicated a carboxylic acid group substituted to the C-1. The hydroxyl group was located at C-4a based on the HMBC correlations from H-3 (δ_H_ 7.25) to C-4a (δ_C_ 154.7). NMR resonances for C-8 and C-10a (δ_C_ 163.6) and for H-5 and H-7 (δ_H_ 6.28) were identical and magnetically equivalent. This observation is in accordance with a symmetrically substituted aromatic ring possessing hydroxyl groups at C-8 as well as C-10a. Therefore, compound **1** was identified as 2-(2,6-dihydroxybenzoyl)-3-hydroxybenzoic acid, and named peniphenone.

Methyl peniphenone (**2**), was isolated as a yellow solid. The HRESIMS (see Figure S7) displayed a negative ion peak at *m*/*z* 287.0557 [M − H]^−^ (calcd. for C_15_H_11_O_6_, 287.0561), corresponding to the molecular formula C_15_H_12_O_6_. There are 10 degrees of unsaturation. The ^1^H NMR (see Figure S8) spectrum showed six aromatic protons containing two AMX spin systems [δ_H_ 7.26 (t, *J* = 8.0 Hz), 7.46 (dd, *J* = 7.8, 0.7 Hz), and 7.03 (d, *J* = 8.1, 0.7 Hz)] and [δ_H_ 7.21 (t, *J* = 8.2 Hz), 6.28 (d, *J* = 8.2 Hz), and 6.28 (d, *J* = 8.2 Hz)], indicating that **2** possessed two fragments of a 1,2,3-trisubstituted benzene ring (Table 1). The ^13^C NMR (see Figure S9) and DEPT spectra gave signals for 15 carbon atoms, including one ketone carbonyl (δ_C_ 203.2, C-9), one ester carbonyl (δ_C_ 168.4, C-11), two aromatic rings, and one methoxy (δ_C_ 52.6, C-12). The spectroscopic information was quite similar to peniphenone (**1**), except for the presence of a methoxy group [δ_H_ 3.69 (s), δ_C_ 52.6]. The HMBC (see Figure S12) correlation of H_3_-12 to C-11 (δ_C_ 168.4) indicated that the methoxy group was substituted to the carbonyl group (δ_C_ 168.4, C-11). Thus, compound **2** was established as the methyl ester analogue of **1**, and named methyl peniphenone.

The immunosuppressive activities (IC_50_ values) of **1**–**9** and the known immunosuppressant azathioprine were calculated against Con A-induced (T cell) and LPS-induced (B cell) proliferations of mouse splenic lymphocytes, as shown in Table 2. The results showed that **1**, **3**, **5**, and **7** possessed potent immunosuppressive activity, while the others were weak. The carboxylic acid group at C-1 (**1**) enhanced the immunosuppressive activity in comparison with **2** bearing a methyl ester group. The immunosuppressive activities of **5** and **7** were stronger than those of **4** and **6**, suggesting that the presence of the hydroxyl group at C-2 is important for the appearance of the immunosuppressive activity of **5** and **7**. However, the methyl or hydroxymethyl groups at C-6 of the xanthones are unlikely to be essential for the immunosuppressive activity (**4** vs. **6** and **5** vs. **7**). It was already known that the suppressive effects of substituted xanthones against the proliferation of human lymphocytes were ascribable to the positions of substituents on the xanthone nucleus [19].

## 3. Experimental Section

### 3.1. General

Melting points were measured on an X-4 micromelting-point apparatus (Cany Precision Instruments Co., Ltd., Shanghai, China) and are uncorrected. UV data were measured on a UV-240 spectrophotometer (Shimadzu, Beijing, China). IR spectra were measured with a Shimadzu IR Affinity-1 Fourier transform infrared spectrophotometer. The NMR data were recorded on a Bruker Avance 400 spectrometer and a Bruker Avance 500 spectrometer (Bruker Bio Spin Corporation, Bellerica, MA, USA), respectively. All chemical shifts (δ) are given in ppm with reference to TMS, and coupling constants (*J*) are given in Hz. LRESIMS spectra were recorded on a Finnigan LCQ-DECA mass spectrometer (Finnigan, Beijing, China). ESIMS spectra were obtained from a Micro mass Q-TOF spectrometer and HRESIMS from a Thermofisher LTQ Orbitrp Elite LC-MS spectrometer. Column chromatography (CC) was performed on silica gel (200–300 mesh, Qingdao Marine Chemical Factory, Qingdao, China) and Sephadex LH-20 (Amersham Pharmacia, Piscataway, NJ, USA). Precoated silica gel plates (Qingdao Huang Hai Chemical Group Co., Qingdao, China; G60, F-254) were used for thin layer chromatography. Semi-preparative HPLC was performed on a Waters Breeze HPLC system using a Phenomenex Luna (Phenomenex, Torrance, CA, USA) C_18_ column (250 × 10 mm, 5 μm), flow rate, 2.0 mL/min.

### 3.2. Fungal Material

The fungal strain used in this study was isolated from the fresh tissue from leaves of *Sonneratia apetala*, which was collected in September 2012 from Zhanjiang Mangrove Nature Reserve in Guangdong Province, China. It was obtained using the standard protocol for the isolation of endophytic microbes [8]. This isolate was identified by Yayue Liu and assigned the accession number ZJ-SY_2_. The sequence data obtained from the fungal strain have been deposited at Gen Bank with accession no. KX890092. A BLAST search result revealed that the sequence was the most similar (99%) to the sequence of *Penicillium* sp. (compared to HQ850365.1 KM222497.1). A voucher strain was deposited in school of chemistry and chemical engineering, Sun Yat-Sen University, Guangzhou, China.

### 3.3. Extraction and Isolation

The fungus *Penicillium* sp. ZJ-SY_2_ was fermented on autoclaved rice solid-substrate medium (sixty 500 mL Erlenmeyer flasks, each containing 50 g rice and 50 mL 3‰ of saline water) for 30 days at 25 °C. Following incubation, the mycelia and solid rice medium were extracted with EtOAc. The organic solvent was filtered and concentrated under reduced pressure to yield 40 g organic extract. The extract was subjected to silica gel CC using gradient elution with petroleum ether-EtOAc from 90:10 to 0:100 (*v*/*v*) to give twelve fractions (Frs.1–12). Fr.2 (596 mg) was further purified by silica gel CC using gradient elution with petroleum ether-EtOAc from 80:20 to 20:80 (*v*/*v*) to afford eight subfractions (Frs.2.1–2.8). Fr.2.5 (20 mg) was further purified by RP-HPLC (75% MeOH in H_2_O) to afford **4** (5.9 mg, *t*_R_ = 16.3 min). Fr.3 (917 mg) was further purified by silica gel CC using gradient elution with petroleum ether-EtOAc from 80:20 to 20:80 (*v*/*v*) to afford seven subfractions (Frs.3.1–3.7). Fr.3.1 (40 mg) was applied to Sephadex LH-20 CC, eluted with CHCl_3_/MeOH (1:1), to obtain eight subfractions (Frs.3.1.1–F3.1.8). Fr.3.1.3 (20 mg) was further purified by silica gel CC using 40% EtOAc-light petroleum to afford **3** (10.9 mg). Fr.3.3 (35 mg) was applied to Sephadex LH-20 CC, eluted with CHCl_3_/MeOH (1:1), to obtain eleven subfractions (Frs.3.3.1–F3.3.11). Fr.3.3.4 (30 mg) was further purified by silica gel CC using 40% EtOAc-light petroleum to afford **8** (28.0 mg) and **9** (20.3 mg). Fr.4 (1.20 g) was further purified by silica gel CC using gradient elution with petroleum ether-EtOAc from 80:20 to 20:80 (*v*/*v*) to afford eleven subfractions (Frs.4.1–4.11). Fr.4.4 (20 mg) was applied to Sephadex LH-20 CC, eluted with MeOH, to obtain six subfractions (Frs.4.4.1–F4.4.6). Fr.4.4.4 (10 mg) was further purified by RP-HPLC (70% MeOH in H_2_O) to afford **6** (10.8 mg, *t*_R_ = 9.0 min) and **5** (1.6 mg, *t*_R_ = 17.6 min). Fr.5 (503 mg) was applied to silica gel CC using 60% EtOAc-petroleum ether to afford seven subfractions (Frs.5.1–5.7). Fr.5.3 was further purified by RP-HPLC (50% MeOH in H_2_O) to afford **1** (3.9 mg, *t*_R_ = 12.5 min) and **2** (5.3 mg, *t*_R_ = 20.9 min). Fr.8 (583 mg) was applied to silica gel CC using 70% EtOAc-petroleum ether to afford seven subfractions (Frs.8.1–8.7). Fr.8.3 was further purified by RP-HPLC (50% MeOH in H_2_O) to afford **7** (2.9 mg, *t*_R_ = 16.5 min).

Peniphenone (**1**): Yellow solid; mp 180–182 °C; UV (MeOH) (λ_max_) (log ε) 205 (4.42), 277 (3.99) nm; IR (KBr) ν_max_ 3342, 2956, 1698, 1594, 1451, 1348, 1297, 1033, 754 cm^−1^; ^1^H and ^13^C NMR spectroscopic data, see Table 2; HRESI-MS (*m*/*z* 273.0401 [M − H]^−^, calcd. for C_14_H_9_O_6_, 273.0405).

Methyl peniphenone (**2**): Yellow solid; mp 168–169 °C; UV (MeOH) (λ_max_) (log ε) 205 (4.43), 273 (3.96) nm; IR (KBr) ν_max_ 3345, 3092, 2933, 1685, 1600, 1450, 1301, 1205, 1020, 931 cm^−1^; ^1^H and ^13^C NMR spectroscopic data, see Table 2; HRESI-MS (*m*/*z* 287.0557 [M − H]^−^, calcd. for C_15_H_11_O_6_, 287.0561).

### 3.4. Immunosuppressive Activity

Compounds (**1**–**9**) were tested for suppressive activity (IC_50_ values) against the proliferation of mouse splenic lymphocytes stimulated with Con-A and LPS using a MTT (3-(4,5-dimethyl-2-thiazolyl)-2,5-diphenyl-2-H-tetrazolium bromide) method according to the literature [20]. This method is based on the formation ratio of 3-(4,5-dimethyl-2-thiazolyl)-2,5-diphenyl-2-H-tetrazolium bromide (MTT)-formazan from exogenous MTT in lymphocytes. A suspension of the splenic lymphocytes from male BALB/c mice (7–11 weeks old, Nippon SLC) was prepared with the FBS/RPMI medium at the concentration of 4.0 × 10^6^ cells/mL. A sample was dissolved in 1.0% EtOH in the FBS/RPMI-medium to prepare a sample solution, 50 μL of which was incubated with 50 μL of the cell suspension, 50 μL of the Con-A (Sigma, St. Louis, MO, USA) or LPS (Sigma, St. Louis, MO, USA) solution (100 μg/mL) in a U-bottom 96-well microtiter plate at 37 °C in a humidified atmosphere of 5% CO_2_ for 48 h. Then, 20 μL of the MTT (FLUCK, 5 mg/mL) solution was added to the culture and incubated for additional 4 h. Next, 100 μL of DMSO was added to the precipitated cells to extract formazan. The absorbance of each DMSO solution was measured at 560 nm with a microplate reader.

## 4. Conclusions

The chemical investigation of a mangrove endophytic fungus *Penicillium* sp. ZJ-SY_2_, isolated from the leaves of *Sonneratia apetala*, led to the discovery of nine polyketides (**1**–**9**). Their structures were established by 1D and 2D NMR spectroscopic data. All isolates were tested for their immunosuppressive activities against Con A-induced (T cell) and LPS-induced (B cell) proliferations of mouse splenic lymphocytes by the MTT method, and compounds **1**, **3**, **5**, and **7** showed moderate immunosuppressive activity. A primary analysis of the structure-activity relationships was discussed.

## Figures and Tables

**Table 1:** ^1^H and ^13^C NMR data (Methanol-*d*_4_, 500/125 MHz, δ ppm, *J* in Hz) of compounds **1** and **2**.

Position	1		2	
	δ_C_	δ_H_ (*J* in Hz)	δ_C_	δ_H_ (*J* in Hz)
1	134.9, C	-	134.7, C	-
2	122.1, CH	7.49, d (7.7)	121.7, CH	7.46, dd (7.8, 0.7)
3	129.7, CH	7.25, t (7.9)	129.9, CH	7.26, t (8.0)
4	120.8, CH	7.01, d (8.1)	121.2, CH	7.03, dd (8.1, 0.7)
4a	154.7, C	-	155.2, C	-
5	108.2, CH	6.28, d (8.2)	108.2, CH	6.28, d (8.2)
6	137.4, CH	7.19, t (8.2)	137.6, CH	7.21, t (8.2)
7	108.2, CH	6.28, d (8.2)	108.2, CH	6.28, d (8.2)
8	163.6, C	-	163.6, C	-
8a	112.9, C	-	112.9, C	-
9	203.6, C	-	203.2, C	-
9a	130.4, C	-	129.5, C	-
10a	163.6, C	-	163.6, C	-
11	169.7, C	-	168.4, C	-
12	-	-	52.6, CH_3_	3.69, s

**Table 2:** Immunosuppressive effects of compounds **1**–**9** and azathioprine on the Con A–induced and LPS-induced proliferations of mouse splenic lymphocytes ^a^.

Compounds	IC_50_ (μg/mL)	
	Con A-Induced	LPS-Induced
**1**	8.1	9.3
**2**	17.5	23.7
**3**	8.2	7.5
**4**	25.7	26.4
**5**	5.9	7.5
**6**	19.2	20.8
**7**	6.5	7.1
**8**	30.1	32.4
**9**	30.8	31.2
Azathioprine	2.7	2.7

^a^ The data shown here were from a representative experiment repeated three times with similar results.

## Supplementary Materials

The supplementary materials are available online at www.mdpi.com/1660-3397/14/12/217/s1, Figure S1: HRESIMS spectrum of **1**, Figure S2: ^1^H NMR spectrum of **1** in CD_3_OD, Figure S3: ^13^C NMR spectrum of **1** in CD_3_OD, Figure S4: ^1^H–^1^H COSY spectrum of **1** in CD_3_OD, Figure S5: HSQC spectrum of **1** in CD_3_OD, Figure S6: HMBC spectrum of **1** in CD_3_OD, Figure S7: HRESIMS spectrum of **2**, Figure S8: ^1^H NMR spectrum of **2** in CD_3_OD, Figure S9: ^13^C NMR spectrum of **2** in CD_3_OD, Figure S10: ^1^H–^1^H COSY spectrum of **2** in CD_3_OD, Figure S11: HSQC spectrum of **2** in CD_3_OD, Figure S12: HMBC spectrum of **2** in CD_3_OD.

## References

[1] Hoffmann M., Rychlewski J., Chrzanowska M., Hermann T. (2001). Mechanism of activation of an immunosuppressive drug: Azathioprine. Quantum chemical study on the reaction of azathioprine with cysteine. J. Am. Chem. Soc..

[2] Linker R.A., Kieseier B.C. (2008). Innovative monoclonal antibodies therapies inmultiplesclerosis. Ther. Adv. Neurol. Disord..

[3] Hauser S.L., Waubant E., Arnold D.L., Vollmer T., Antel J., Fox R.J., Bar-Or A., Panzara M., Sarkar N., Agarwal S. (2008). B-cell depletion with rituximab in relapsing remitting multiple sclerosis. N. Engl. J. Med..

[4] García-Carrasco M., Jiménez-Hernández M., Escárcega R.O. (2009). Use of rituximab in patients with systemic lupusery thematosus: An update. Autoimmun. Rev..

[5] Rateb M.E., Ebel R. (2011). Secondary metabolites of fungi from marine habitats. Nat. Prod. Rep..

[6] Chen S., Chen D., Cai R., Cui H., Long Y., Lu Y., Li C., She Z. (2016). Cytotoxic and antibacterial preussomerins from the mangrove endophytic fungus *Lasiodiplodia theobromae* ZJ-HQ1. J. Nat. Prod..

[7] Liu Z., Chen Y., Chen S., Liu Y., Lu Y., Chen D., Lin Y., Huang X., She Z. (2016). Aspterpenacids A and B, two sesterterpenoids from a mangrove endophytic fungus *Aspergillus terreus* H010. Org. Lett..

[8] Chen S., Liu Y., Liu Z., Cai R., Lu Y., Huang X., She Z. (2016). Isocoumarins and benzofurans from the mangrove endophytic fungus *Talaromyces amestolkiae* possess α-glucosidase inhibitory and antibacterial activities. RSC Adv..

[9] Liu Y., Chen S., Liu Z., Lu Y., Xia G., Liu H., He L., She Z. (2015). Bioactive metabolites from mangrove endophytic fungus *Aspergillus* sp. 16-5B. Mar. Drugs.

[10] Chen S., Liu Z., Liu Y., Lu Y., He L., She Z. (2015). New depsidones and isoindolinones from the mangrove endophytic fungus *Meyerozyma guilliermondii* (HZ-Y2) isolated from the South China Sea. Beilstein J. Org. Chem..

[11] Liu Z., Xia G., Chen S., Liu Y., Li H., She Z. (2014). Eurothiocin A and B, sulfur-containing benzofurans from a soft coral-derived fungus *Eurotium rubrum* SH-823. Mar. Drugs.

[12] Huang X., Huang H., Li H., Sun X., Huang H., Lu Y., Lin Y., Long Y., She Z. (2013). Asperterpenoid A, a new sesterterpenoid as an inhibitor of mycobacterium tuberculosis protein tyrosine phosphatase B from the culture of *Aspergillus* sp. 16-5c. Org. Lett..

[13] Xiao Z., Huang H., Shao C., Xia X., Ma L., Huang X., Lu Y., Lin Y., Long Y., She Z. (2013). Asperterpenols A and B, new sesterterpenoids isolated from a mangrove endophytic fungus *Aspergillus* sp. 085242. Org. Lett..

[14] Wang Y., Zheng Z., Liu S., Zhang H., Li E., Guo L., Che Y. (2010). Oxepinochromenones, furochromenone, and their putative precursors from the endolichenic fungus *Coniochaeta* sp.. J. Nat. Prod..

[15] Kongkiat T., Vatcharin R., Morakot K., Souwalak P., Nongporn H., Sita P., Jariya S. (2011). Sesquiterpene and xanthone derivatives from the sea fan-derived fungus *Aspergillus sydowii* PSU-F154. J. Nat. Prod..

[16] Yao Q., Wang J., Zhang X., Nong X., Xu X., Qi S. (2014). Cytotoxic polyketides from the deep-sea-derived fungus *Engyodontium album* DFFSCS021. Mar. Drugs.

[17] Hamasaki T., Sato Y., Hatsuda Y. (1975). Structure of sydowinin A, sydowinin B, and sydowinol, metabolites from *Aspergillus sydowi*. Agric. Biol. Chem..

[18] Xia M., Cui C., Li C., Wu C., Peng J., Li D. (2015). Rare chromones from a fungal mutant of the marine-derived *Penicillium purpurogenum* G59. Mar. Drugs.

[19] Pedro M., Cerqueira F., Sousa M.E., Nascimento M.S.J., Pinto M. (2002). Xanthones as inhibitors of growth of human cancer cell lines and their effects on the proliferation of human lymphocytes in vitro. Bioorgan. Med. Chem..

[20] Fujimoto H., Asai T., Kim Y., Ishibashi M. (2006). Nine constituents including six xanthone-related compounds isolated from two ascomycetes, gelasinospora santi-florii and emericella quadrilineata, found in a screening study focused on immunomodulatory activity. Chem. Pharm. Bull..

