# Supplementary material for: Polyketides with Immunosuppressive Activities from Mangrove Endophytic Fungus Penicillium sp. ZJ-SY2
Source: Mar Drugs. 2016 Nov 25;14(12):217. doi: 10.3390/md14120217 (PMC5192454; doi:10.3390/md14120217)
Supplement: Supplementary file 1 [file marinedrugs-14-00217-s001.docx]

**Supplementary Materials: Polyketides with Immunosuppressive Activities from Mangrove Endophytic Fungus *Penicillium* sp. ZJ-SY_2_**

# Hongju Liu, Senhua Chen, Weiyang Liu, Yayue Liu, Xishan Huang and Zhigang She

##

## **Figure S1.** HREIMS spectrum of **1**.

##
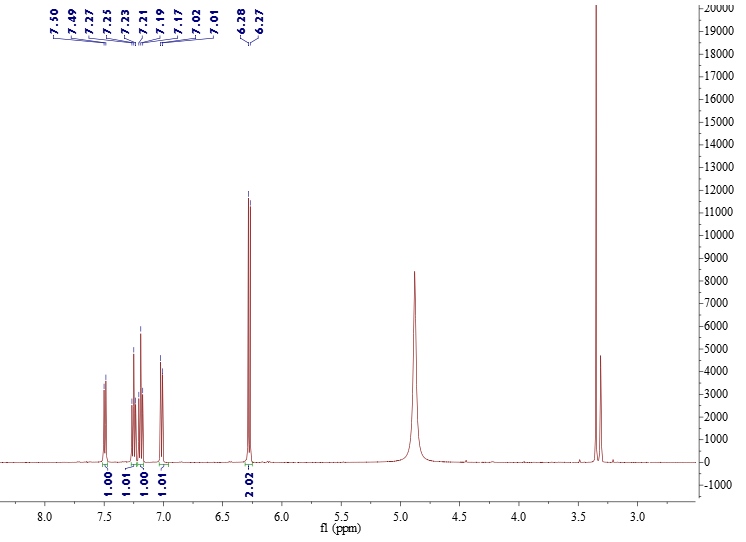


## **Figure S2.** ^1^H NMR spectrum of **1** in CD_3_OD.

##
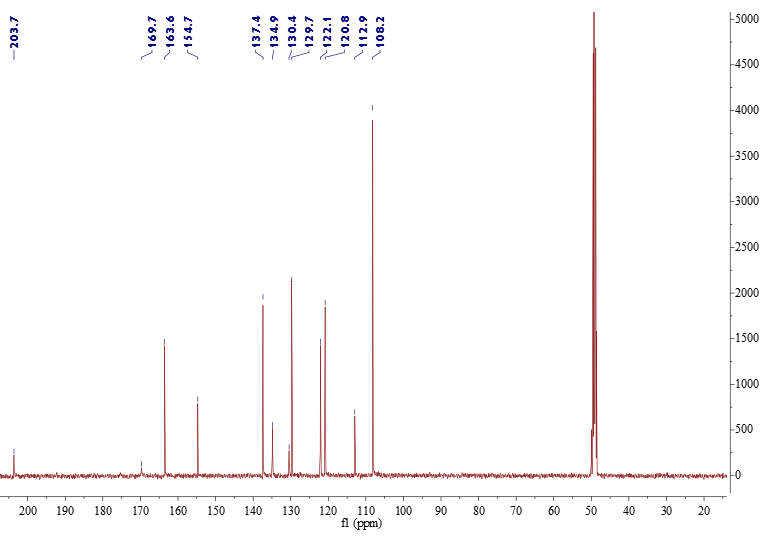


## **Figure S3.** ^13^C NMR spectrum of **1** in CD_3_OD.

##
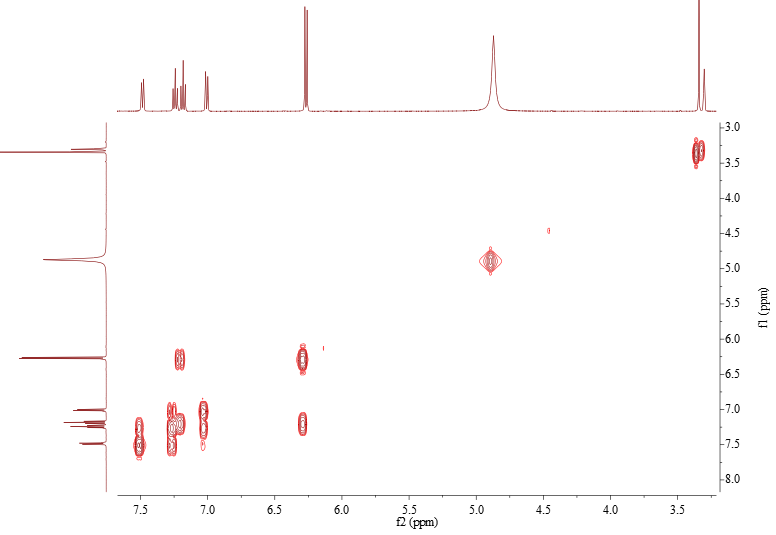


## **Figure S4.** ^1^H–^1^H COSY spectrum of **1** in CD_3_OD.

##
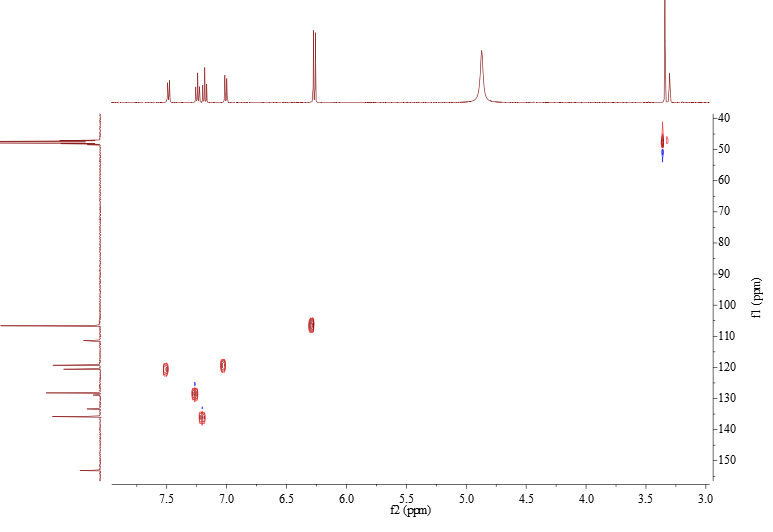


## **Figure S5.** HSQC spectrum of **1** in CD_3_OD.

##
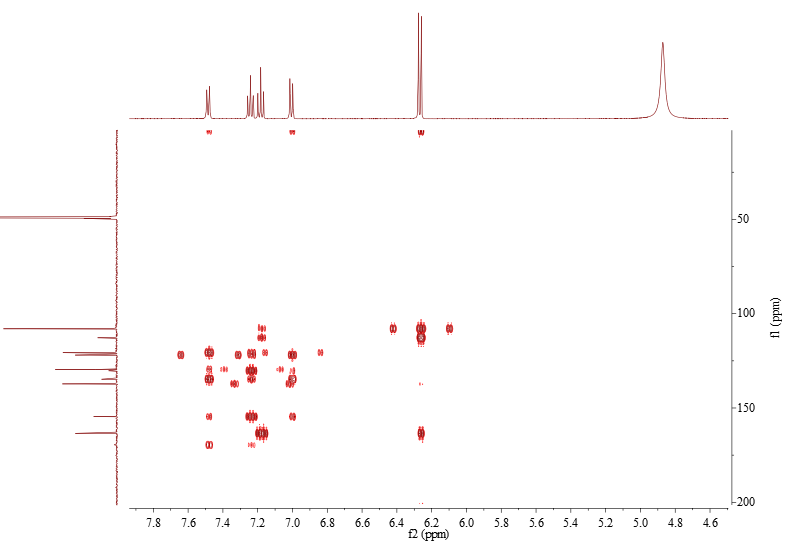


## **Figure S6.** HMBC spectrum of **1** in CD_3_OD.

##

## **Figure S7.** HREIMS spectrum of **2**.

##
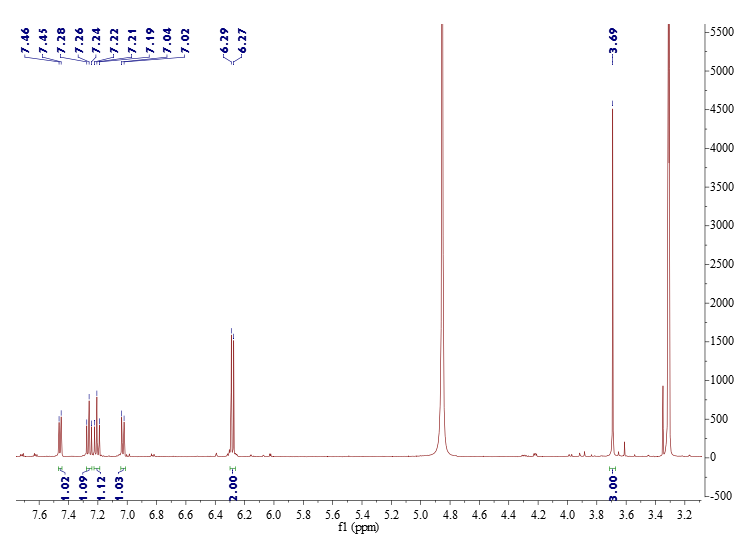


## **Figure S8.** ^1^H NMR spectrum of **2** in CDCl_3_.

##
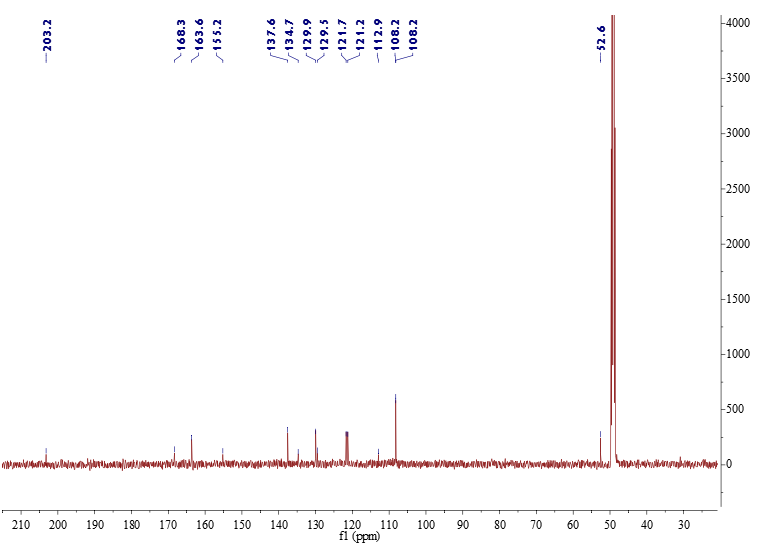


## **Figure S9.** ^13^C NMR spectrum of **2** in CDCl_3_.

##
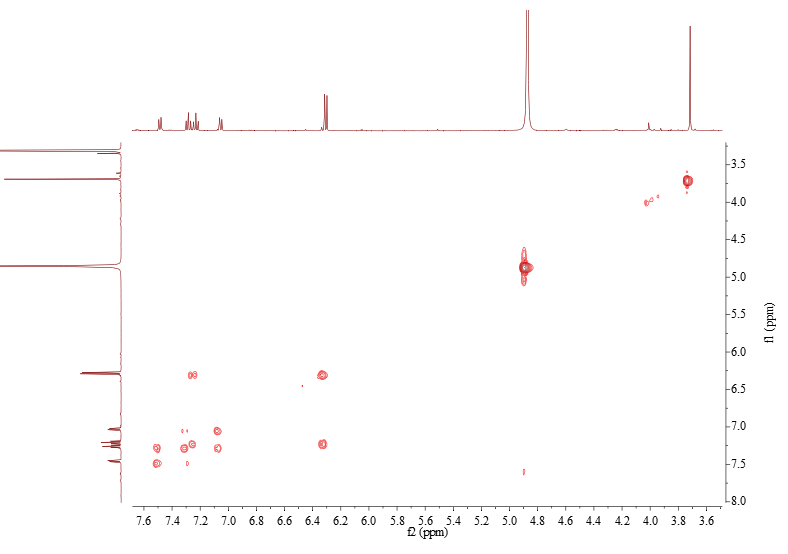


## **Figure S10.** ^1^H–^1^H COSY spectrum of **2** in CDCl_3_.

##
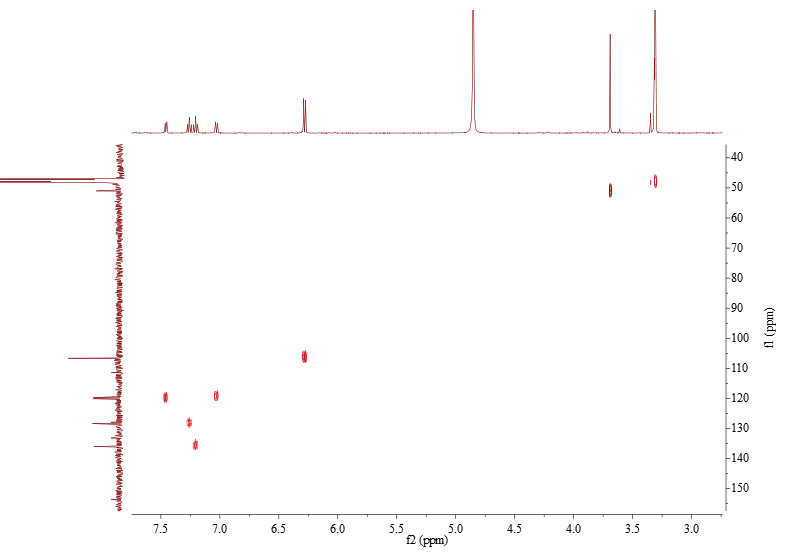


## **Figure S11.** HSQC spectrum of **2** in CDCl_3_.

##
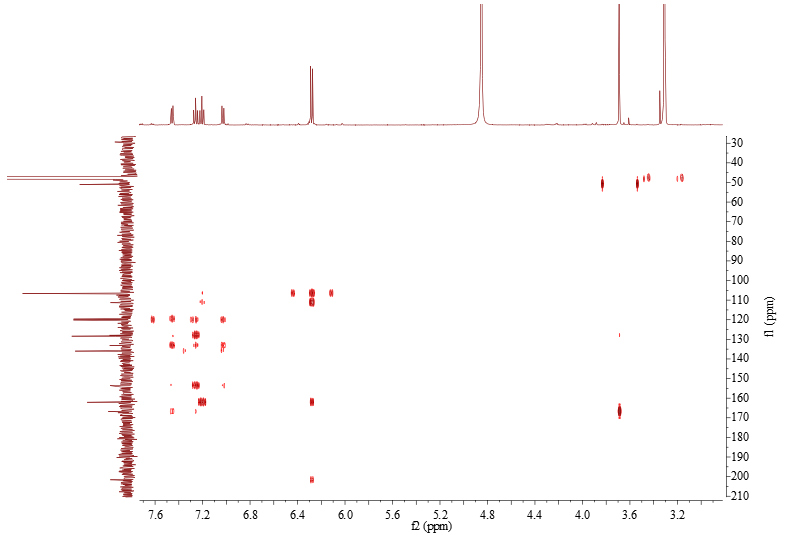


## **Figure S12.** HMBC spectrum of **2** in CDCl_3_.
